# Exploring the ability of vocal biomarkers in distinguishing depression from bipolar disorder, schizophrenia, and healthy controls

**DOI:** 10.3389/fpsyt.2023.1079448

**Published:** 2023-07-20

**Authors:** Wei Pan, Fusong Deng, Xianbin Wang, Bowen Hang, Wenwei Zhou, Tingshao Zhu

**Affiliations:** ^1^Key Laboratory of Adolescent Cyberpsychology and Behavior (CCNU), Ministry of Education, Wuhan, China; ^2^School of Psychology, Central China Normal University, Wuhan, China; ^3^Key Laboratory of Human Development and Mental Health of Hubei Province, Wuhan, China; ^4^Wuhan Wuchang Hospital, Wuchang Hospital Affiliated to Wuhan University of Science and Technology, Wuhan, China; ^5^Institute of Psychology, Chinese Academy of Sciences, Beijing, China; ^6^CAS Key Laboratory of Behavioral Science, Institute of Psychology, Chinese Academy of Sciences, Beijing, China

**Keywords:** depression, healthy controls, schizophrenia, bipolar disorder, i-vectors, logistic regression MFCCs

## Abstract

**Background:**

Vocal features have been exploited to distinguish depression from healthy controls. While there have been some claims for success, the degree to which changes in vocal features are specific to depression has not been systematically studied. Hence, we examined the performances of vocal features in differentiating depression from bipolar disorder (BD), schizophrenia and healthy controls, as well as pairwise classifications for the three disorders.

**Methods:**

We sampled 32 bipolar disorder patients, 106 depression patients, 114 healthy controls, and 20 schizophrenia patients. We extracted i-vectors from Mel-frequency cepstrum coefficients (MFCCs), and built logistic regression models with ridge regularization and 5-fold cross-validation on the training set, then applied models to the test set. There were seven classification tasks: any disorder versus healthy controls; depression versus healthy controls; BD versus healthy controls; schizophrenia versus healthy controls; depression versus BD; depression versus schizophrenia; BD versus schizophrenia.

**Results:**

The area under curve (AUC) score for classifying depression and bipolar disorder was 0.5 (*F*-*score* = 0.44). For other comparisons, the AUC scores ranged from 0.75 to 0.92, and the *F*-*scores* ranged from 0.73 to 0.91. The model performance (AUC) of classifying depression and bipolar disorder was significantly worse than that of classifying bipolar disorder and schizophrenia (corrected *p* < 0.05). While there were no significant differences in the remaining pairwise comparisons of the 7 classification tasks.

**Conclusion:**

Vocal features showed discriminatory potential in classifying depression and the healthy controls, as well as between depression and other mental disorders. Future research should systematically examine the mechanisms of voice features in distinguishing depression with other mental disorders and develop more sophisticated machine learning models so that voice can assist clinical diagnosis better.

## Introduction

The identification and diagnosis of depression through clinical interviews are often slow and unreliable (1–4). About half of cases go unrecognized: in a meta-analysis of 41 studies, recognition accuracy of depression by general practitioners was 47.3% (5). Therefore, accurate and fast ways to identify cases of depression will have major clinical benefits.

Novel applications of computational methods are making some inroads into this problem. For example, a review of 14 studies indicated that both sensitivity and specificity of diagnostic performance of deep learning models were higher than that of health-care professionals (6). In the last decade, there have been interests in the ability of exploiting vocal biomarkers to identify depression with machine learning methods to investigate whether voice can be used as an auxiliary tool to assist clinical diagnosis (7–11). Previous research mainly focused on examining the ability of vocal features in classifying individuals with depression and healthy population (12–17), and *F*-measure of relevant classifiers reached 0.9 (12). These findings suggest that vocal biomarkers may have discriminatory potential in identifying depression. While the differential diagnosis is complicated by the presence of other mental disorders.

The prevalence of mental disorders, according to a study in China, showed that the weighted prevalence of any disorder (excluding dementia) was 16.6% (95% CI 13.0–20.2) during the 12 months before the interview (18). Clinically, the psychiatric diagnosis necessitates distinguishing depressed individuals not only from the healthy ones, but also from other mental illnesses with similar mood symptoms or similar voice patterns. It needs to be stressed that voice conveys emotion related information. In the field of affective computing, voice features were shown their abilities in recognizing different kinds of emotions (19, 20). Both bipolar disorder (BD) and schizophrenia exhibit symptoms comparable to depression. According to the Diagnostic and Statistical Manual of Mental Disorders, Fourth Edition (DSM-4) (21), BD involves both depressive and manic episodes, while schizophrenia patients with negative symptoms report anhedonia (loss of ability to experience pleasure). The specificity of alterations in vocal characteristics to depression has not been rigorously investigated.

Variations in vocal features have been observed in schizophrenia and BD patients. Acoustic analyses of speech in schizophrenia have revealed subtle aberrancies in pitch variability associated with flat affect, as well as more pronounced deviations in properties such as percentage of speaking time, speech rate, and pause duration that correlate with symptoms of alogia and blunted affect (7). Espinola and her colleagues (22) constructed classification models based on speech samples from 31 individuals (20 of whom had a prior diagnosis of schizophrenia and 11 healthy controls). The classifiers attained an accuracy of 91.76% in distinguishing between groups. Regarding BD, studies have utilized vocal features to predict patients’ emotional states (e.g., depressive, manic, mixed states). Classification analyses yielded an area under curve (AUC) of 0.89 (9, 10). Another study investigated whether vocal features acquired via verbal fluency tasks could accurately differentiate mixed states in BD using machine learning methods. And results showed that for depressive versus mixed depressive episodes, the *F*-measure was 0.86, while for hypomanic versus mixed hypomanic episodes, the *F*-measure was 0.75 (23). These studies showed that voice features may also be informative for other psychiatric diagnosis. It should be noted that several studies have examined the utility of vocal features in developing classifiers for several mental disorders. A study (24) employed polytomous logistic regression analysis of vocal features to discriminate among healthy controls (*n* = 23), individuals with bipolar disorder (*n* = 8), and those with major depressive disorder (*n* = 14). The model attained 90.79% accuracy in classifying participants into the three diagnostic groups. Another study (25) proposed a methodology to support the diagnosis of several mental disorders using vocal acoustic analysis and machine learning. The results showed that random forests with 300 trees achieved the best classification performance (75.27% for accuracy) for the simultaneous detection of major depressive disorder (26), schizophrenia (20), BD (15), generalized anxiety disorder (4), and healthy controls (12). However, the datasets of above two studies were imbalanced for each group. The imbalanced dataset problems become more complicated in multi-class imbalanced classification tasks, in which there may be multiple minority and majority classes that cause skewed data distribution. And machine learning algorithms tend to favor the majority class samples, hence damaging the multi-classification results (27, 28). Moreover, extensive comparisons between mental disorders and healthy controls may offer more information about the effectiveness of voice for clinically complex differential diagnosis.

Various speech features are indicative of depression. Mel-frequency cepstrum coefficients (MFCCs) constitute the most prevalent vocal features employed in speech recognition systems and psychiatric condition classification models (26). MFCCs are obtained by extracting frequency spectral features of the speech signal using the short-time power spectrum, mapping these features onto the Mel scale to better present auditory characteristics, and then obtaining MFCC coefficients through cepstrum analysis that can characterize the speech envelope (12, 29). Multiple studies have demonstrated the utility of MFCCs in developing classification models for depression detection (30–32). For example, Di et al. (29) employed MFCCs to classify major depression patients and healthy individuals, area under curve (AUC) reached 0.8.

The identity vector (i-vector) approach, grounded within the total variability framework, represents the state-of-the-art technique for speaker verification (12, 29, 32, 33). The total variability framework offers an effective means of capturing speaker- and channel-related variability in a low dimensional subspace. i-vectors are highly informative for encoding cepstral variability. Classification models based on i-vectors demonstrated capacity for identifying depression with high accuracy. For instance, prior work found i-vector based model outperformed a baseline model defined by KL-means supervectors (32). Nasir and his colleagues (33) used i-vectors to investigate various audio and visual features for classification, reporting high accuracy with i-vector modeling of MFCC features. Indeed, one study demonstrated a 40% improvement in predictive accuracy (*F*-*score*) with the i-vector methodology (12). And Di et al. (29) observed a 14% enhancement in model performance (AUC) with i-vectors relative to MFCCs alone. Although the participant cohorts were exclusively female in both studies, the results demonstrated the promise of i-vectors for enhancing the accuracy of machine learning models for depression classification.

The objective of this study was to evaluate the efficacy of vocal features as differential diagnostic markers for depression compared to other psychiatric disorders. Three binary classification paradigms were employed in total: (1) the capacity of voice features to distinguish any psychiatric condition (depression, bipolar disorder, schizophrenia) versus healthy controls at baseline; (2) the ability of voice features to differentiate a specific psychiatric illness from healthy controls; (3) the capability of vocal features to distinguish between discrete psychiatric disorders in a pairwise manner. Among these paradigms, the first one was served as a baseline to determine whether the dataset achieved performance commensurate with existing research, as well as a benchmark for model performance under other framework conditions. The second and third paradigms were employed to systematically evaluate the capacity of vocal characteristics to distinguish between case and control groups.

## Methods

### Participants

All participants were randomly recruited. All participants were Chinese aged 18 to 59 years. A diagnosis of primary psychiatric illness was established for all patients using the Diagnostic and Statistical Manual of Mental Disorders (DSM-IV) (21) by psychiatrists. Participants clustered into four categories based on diagnosis: healthy controls, depression, BD and schizophrenia. Clinical staging was further specified: depression subjects were actively symptomatic, BD patients were euthymic, and schizophrenia patients were in remission. Healthy controls were openly recruited. Patients with comorbid psychiatric conditions were excluded for all diagnostic categories. And the general exclusion criteria across all participants were: physical illnesses, pregnancy and lactating, substance abuse within 12 months. Demographic variables were age, gender and education level. See Table 1.

**Table 1:** Demographic information about each group.

Groups	Gender	Age	Education	Occupation
Health	58 males	34.88 ± 10.54	7.78 ± 2.60	4.13 ± 3.48
	57 females	34.73 ± 9.69	7.47 ± 2.53	3.08 ± 3.13
Depression	53 males	32.67 ± 8.62	6.70 ± 2.36	2.34 ± 2.76
	70 females	34.31 ± 11.22	7.69 ± 2.25	3.32 ± 3.20
Bipolar	16 males	30.06 ± 10.46	6.75 ± 2.61	2.55 ± 2.74
	21 females	33.10 ± 10.59	7.02 ± 2.16	4.93 ± 3.14
Schizophrenia	10 males	27.86 ± 7.14	6.81 ± 2.33	3.84 ± 3.34
	10 females	30.81 ± 7.61	6.41 ± 2.28	4.64 ± 3.41

### Measures

Four vocal tasks were employed for data collection: video watching (VW), text reading (TR), question answering (QA), picture description (PD). Each task incorporated positive, negative, neutral emotional primes to comprehensively represent existing research paradigms (34–38). In VW, participants viewed video clips then described the most memorable scenes or figures. For QA, participants provided spoken answers to nine questions (three questions/emotion), e.g., “Please share your most wonderful experience and describe it in detail”. In TR, participants read three 140-word paragraphs aloud. For PD, participants described facial expressions and image content from the Chinese Facial Affective Picture System and the Chinese Affective Picture System (three facial affective pictures and three affective pictures for three emotion primes), respectively. Twenty one voice recordings were collected from each participant. The emotional priming effects of these tasks were validated in previous research (37, 38). Research also indicates that this dataset affords stable prediction accuracies across emotions and tasks (15, 35, 37, 38).

All participants were seated 1 m from a 21-inch monitor. Instructions were displayed on-screen. Speech was recorded 50 cm distant using a professional condenser microphone (Neumann TLM102, Germany) and a voice recorder (RME Fireface UCX, Germany). The experimenter controlled recording initiation and termination for each participant to exclude the experimenter’s speech from recordings. Participants were asked to complete all tasks in random order. Ambient noise was under 60 dB. Recordings less than 10 s were excluded. Recording duration details were displayed in Table 2. The speech was Mandarin Chinese. Recordings were collected with a sampling rate of 44.1 kHz and 24-bit. Informed consent was obtained in writing pre-experiment. This study was part of a national project and was approved by the Institutional Review Board (IRB) of Institute of Psychology, Chinese Academy of Sciences.

**Table 2:** Duration descriptions of voice recordings in each group(s).

Groups	Min	Max	M ± SD
Health	10	188	27.8 ± 17.99
Depression	10	164	29.4 ± 17.59
Bipolar	10	156	30.77 ± 17.74
Schizophrenia	10	149	30.15 ± 20.83

### Data analysis

#### Preprocessing

A total of seven classification tasks were employed: any disorder versus healthy controls; depression versus healthy controls; BD versus healthy controls; schizophrenia versus healthy controls; depression versus BD; depression versus schizophrenia; BD versus schizophrenia (AH, DH, BH, SH, DB, DS, BS). In each task, either the mental disorder group or depression group was designated as the case group, with the other constituting the control group.

For model building, the data were randomly split into training set (70%) and test set (30%). Given the small sample size, group differences in demographics were assessed using permutation test (a permutation t test), a nonparametric method suitable for small samples with unknown distribution. Many parametric tests have their corresponding permutation test versions employing the same test statistic but deriving *p*-values from the sample-specific permutation distribution of that statistic, rather than from the theoretical distribution derived from the parametric assumption (39).

Previous research indicates demographic factors confound the detection of depression from voice features (15). It was therefore critical to control for demographics to isolate the role of vocal features. Age, gender and education differences between groups were evaluated in the training set. Owing to the small, uneven group sizes, matching was performed twice: (1) matched gender within categories through exact matching under the guidance of random sampling (a coarsened matching method for binary variables) (40) to match the number of males and females according to the smaller number within the group by the sample function in R (41). For example, 16 females were randomly selected in the BD group. After matching, there were 32 BD patients; 106 depression patients, 114 healthy ones, and 20 schizophrenia patients with unbiased sex ratios in each category. (2) Case-control matching within each classification task, e.g., 32 BD patients and 32 healthy controls in the model of classifying the BD versus healthy control task. Ultimately, the demographics of cases and controls were preliminarily balanced for each task.

### Mel frequency cepstral coefficients

Mel Frequency Cepstral Coefficients (MFCCs) were extracted by MATLAB R2020b (42) with a window size of 25 ms, a window shift of 10 ms, a pre-emphasis filter with coefficient 0.97, and a sinusoidal lifter with coefficient 22 (12). A filter bank with 23 filters was used and 13 coefficients were extracted. Utterances were downsampled to 8 kHz before feature extraction. The first and second derivatives of MFCCs were also extracted.

MFCCs were extracted for each vocal task from every participant. For each participant, the mean values across the 21 tasks were calculated to streamline modeling. And prior research validates the consistent efficacy of vocal features across vocal tasks (15, 35, 37, 38).

### I-vector extraction

The i-vector extraction formula is represented as follows:


(1) M=m+Tv

where *m* is the mean super-vector of the Universal Background Model (UBM). UBM representing the feature distribution of the acoustic space, is adapted to a set of given speech frames to estimate utterance-dependent Gaussian Mixture Models parameters. *M* is the mean-centered super-vector of the speech utterance derived using the 0th and 1st order Baum–Welch statistics. *v* is the i-vector, the representation of a speech utterance (43).

Twenty i-vectors were derived for each participant. All i-vectors underwent quantile normalization. The training data was then used for constructing logistic regression models. i-vectors extraction was performed using the Kaldi toolkit (44).

### Logistic regression

i-vectors were subjected to logistic regression on the training set using R (41). Logistic regression employed the Glmnet method (45) with ridge regularization and 5-fold cross validation. The resultant models were then applied to the test sets for each classification task.

### Model building

To evaluate the classification ability of voice features for differential diagnosis, logistic regression models were constructed for the seven binary classification tasks. First, we examined the classification ability of voice features in distinguishing between the healthy controls and those with any mental disorder. Second, we examined model performances in separately classifying the healthy controls and each discrete clinical group. Third, model performances were evaluated in pairwise classification among the three disorders (depression versus BD; depression versus schizophrenia; BD versus schizophrenia).

## Results

### Descriptive statistics

Age differences were tested. As shown in Table 3, cases and controls for all classification tasks were matched on relevant variables, except for the schizophrenia versus healthy control task. Propensity score matching (46) was therefore conducted to re-match the schizophrenia and the healthy control group. The case group and control group for this task were then balanced, as detailed in Table 4.

**Table 3:** *t*-tests of age difference for each classification task.

Tasks	Groups	M ± SD	*t*	*p*
AH^a^	Any disorder	32.35 ± 9.82	−1.42	0.16
	Healthy controls	34.48 ± 10.63		
DH	Depression	33.76 ± 10.23	−0.4	0.69
	Healthy controls	34.43 ± 10.48		
BH	BD	29.68 ± 8.99	−1.85	0.07
	Healthy controls	34.86 ± 9.03		
SH	Schizophrenia	29.07 ± 7.42	−2.11	0.03^*^
	Healthy controls	37.14 ± 7.42		
DB	Depression	34.18 ± 9.67	1.57	0.12
	BD	29.68 ± 8.99		
DS	Depression	33.86 ± 9.49	1.45	0.15
	Schizophrenia	29.07 ± 7.42		
BS	BD	30.00 ± 7.75	0.33	0.77
	Schizophrenia	29.07 ± 7.42		

a AH, any disorder versus healthy controls; DH, depression versus healthy controls; BH, BD versus healthy controls; SH, schizophrenia versus healthy controls; DB, depression versus BD; DS, depression versus schizophrenia; BS, BD versus schizophrenia. Similarly hereinafter.

^*^p < 0.05.

**Table 4:** *t*-test after propensity score matching for SH task.

Tasks	Groups	M ± SD	*t*	*p*
S_H	Schizophrenia	28.79 ± 8.15	−0.74	0.48
	Healthy controls	31.14 ± 8.74		

Permutation tests were conducted for both gender and education. No significant differences either in gender or education across different tasks. See Tables 5, 6.

**Table 5:** Permutation tests on gender for each classification task.

Tasks	Groups	Gender		*ϰ* ^2^	*p*
		Male	Female		
AH	Any disorder	55	55	0	1
	Healthy controls	40	40		
DH	Depression	37	37	0	1
	Healthy controls	37	37		
BH	BD	11	11	0	1
	Healthy controls	11	11		
SH	Schizophrenia	7	7	0	1
	Healthy controls	7	7		
DB	Depression	11	11	0	1
	BD	11	11		
DS	Depression	7	7	0	1
	Schizophrenia	7	7		
BS	BD	7	7	0	1
	Schizophrenia	7	7		

**Table 6:** Permutation tests on education for each classification task.

Tasks	Groups	Education		*ϰ* ^2^	*p*
		High school level and below	Undergraduate and above		
AH	Any disorder	46	64	0.18	0.76
	Healthy controls	31	49		
DH	Depression	29	45	0.03	1
	Healthy controls	28	46		
BH	BD	9	13	0	1
	Healthy controls	9	13		
SH	Schizophrenia	6	8	1.29	0.45
	Healthy controls	9	5		
DB	Depression	10	12	0.09	1
	BD	9	13		
DS	Depression	5	9	1.29	0.45
	Schizophrenia	8	6		
BS	BD	7	7	0.14	1
	Schizophrenia	8	6		

Following matching, duration differences between groups were evaluated for each classification task. The mean value of 21 recording durations was first computed for each participant across all experimental tasks. Difference tests were then conducted between groups for each classification task. Results showed that there were no significant differences between groups for any classification task. See Table 7.

**Table 7:** Duration differences between groups for each classification task.

Tasks	Groups	M ± SD	*t*	*p*
AH	Any disorder	30.47 ± 8.84	1.53	0.13
	Healthy controls	28.65 ± 7.44		
DH	Depression	30.62 ± 7.80	1.59	0.12
	Healthy controls	28.22 ± 10.36		
BH	BD	30.09 ± 9.93	0.63	0.53
	Healthy controls	28.42 ± 7.81		
SH	Schizophrenia	29.22 ± 6.66	0.14	0.89
	Healthy controls	28.82 ± 8.65		
DB	Depression	30.94 ± 7.68	0.57	0.57
	BD	29.58 ± 8.17		
DS	Depression	31.21 ± 7.22	0.77	0.45
	Schizophrenia	29.17 ± 6.60		
BS	BD	29.54 ± 10.01	0.42	0.68
	Schizophrenia	28.22 ± 5.59		

### Classification

Model performance metrics for different tasks were summarized in Table 8. For the general ability of vocal features to classify healthy versus any clinical group (AH task), the *F*-*score* was 0.82, AUC (area under curve) was 0.79. In distinguishing specific disorders from healthy controls, results showed: for DH task, *F*-*score* = 0.78, AUC = 0.77; for BH task, *F*-*score* = 0.80, AUC = 0.80; for SH task, *F*-*score* = 0.73, AUC = 0.75. To further examine the ability of voice features on pairwise classifications among the three mental disorders, DB, DS and BS tasks were performed. Results showed: for DB task *F*-*score* = 0.44, AUC = 0.50; for DS task, *F*-*score* = 0.83, AUC = 0.83; for BS task, *F*-*score* = 0.91, AUC = 0.92.

**Table 8:** Results on 7 classification tasks with the i-vector framework.

Tasks	Sensitivity	Specificity	Accuracy	Precision	Recall	*F*-score	AUC
AH	0.81	0.76	0.79	0.83	0.81	0.82	0.79
DH	0.81	0.72	0.77	0.74	0.81	0.78	0.77
BH	0.80	0.80	0.80	0.80	0.80	0.80	0.80
SH	0.67	0.83	0.75	0.80	0.67	0.73	0.75
DB	0.40	0.60	0.50	0.50	0.4	0.44	0.50
DS	0.83	0.83	0.83	0.83	0.83	0.83	0.83
BS	0.83	1.00	0.92	1.00	0.83	0.91	0.92

We also compared model performances for all classification tasks. Following Bonferroni correction (*n* = 6), the only significant difference was that the depression versus BD model (DB task) showed worse performance (lower AUC) than the BD versus schizophrenia model (BS task; *p* < 0.05), as detailed in Table 9.

**Table 9:** Pairwise comparisons of model performances for the 7 classification tasks.

Model comparisons	DeLong’s test	df	*p*	Corrected *p*
AH_DH	0.32	134.67	0.75	4.50
AH_BH	−0.11	29.02	0.91	5.46
AH_SH	0.27	13.78	0.79	4.74
AH_DB	2.32	25.54	0.03	0.18
AH_DS	−0.35	14.66	0.73	4.38
AH_BS	−1.34	18.71	0.20	1.20
DH_BH	−0.32	32.18	0.75	4.50
DH_SH	0.11	14.70	0.92	5.52
DH_DB	2.09	27.64	0.05	0.28
DH_DS	−0.52	15.88	0.61	3.66
DH_BS	−1.53	21.29	0.14	0.84
BH_SH	0.30	21.48	0.76	4.56
BH_DB	2.01	36.64	0.05	0.31
BH_DS	−0.22	23.92	0.83	4.98
BH_BS	−0.93	29.35	0.36	2.16
SH_DB	1.41	25.27	0.17	1.02
SH_DS	−0.47	21.63	0.65	3.90
SH_BS	−1.05	18.37	0.31	1.86
DB_DS	−2.02	27.56	0.05	0.32
DB_BS	−2.93	29.93	0.01	0.04^*^
DS_BS	−0.58	19.80	0.57	3.42

*p < 0.05.

## Discussion

In this research, we investigated the ability of vocal biomarkers to classify various health conditions. Following matching, descriptive statistics showed no differences in demographic variables between the case and control groups for any task, addressing potential threats to validity (15). Logistic regression models based on MFCC-derived i-vectors were developed for all classification tasks. Results indicated vocal features may assist differential diagnosis of depression, albeit with varying degrees of effectiveness across classification tasks.

The AH model examined the baseline ability of vocal features to distinguish clinical from healthy groups. Classifying the healthy group and the any-disorder group yielded an *F*-*score* of 0.82 and AUC of 0.79, demonstrating vocal features can distinguish mental disorders from the health.

Furthermore, the DH, BH, and SH models investigated the ability of voice features in distinguishing specific disorders from health. For DH, BH, and SH tasks, *F*-*scores* ranged from 0.73 to 0.80, AUC scores from 0.75 to 0.80. Model comparisons showed there were no significant differences in the pairwise comparisons among the AH and the three mental illnesses versus healthy control classification tasks. Our results about DH are consistent with the existing findings (8, 12, 15, 47–50). For example, a previous study examined the significance of the association between voice features and depression using binary logistic regression, and the actual classification ability of voice features on depression using machine learning method, results showed that the contribution effect of voice features reached 35.65% (Nagelkerke’s *R*^2^), further classification model achieved 81% of *F*-measure (15). We set this classification model as another baseline for the reference of further classification. The results of BH and SH classification models are also consistent with existing studies. For instance, one study investigated whether voice features from naturalistic phone calls could discriminate between BD and healthy control individuals, results showed that compared to the control group, BD was classified with a 0.79 *sensitivity* and 0.76 AUC (51). For schizophrenia detection, Tahir et al. (52) classified schizophrenia patients and healthy controls with multilayer perceptron and the accuracy achieved 81.3%. It was also suggested that speech abnormalities, related to flat affect and alogia, have been a hallmark feature of schizophrenia, and are often associated with core negative symptoms and social impairment (7). Our results indicate that MFCCs capture information distinguishing depression, BD and schizophrenia from health.

The DB, DS, and BS models further evaluated the performances of voice features on pairwise classifications among the three mental disorders. DS (*F*-*score* = 0.83; AUC = 0.83) and BS (*F*-*score* = 0.91; AUC = 0.92) models showed promise. The BS model had the highest *F*-*score* and AUC across all seven models. However, the DB model performed the worst (*F*-*score* = 0.44), with an AUC score of 0.50 indicating voice features barely distinguished depression and BD. Further pairwise model comparisons showed no significant differences among the AH, DH, BH, SH, DS, and BS models. However, DB model performance was significantly worse than that of BS. Results indicate that voice features could help distinguish depression from disorders with similar mood symptoms.

Differing symptoms and vocal characteristics likely explain the results of differential diagnosis. For example, depression is associated with monotonous, hoarse, breathy or slurred speech reflecting anhedonia and sadness (53–58). Schizophrenia is linked to poverty of speech, increased pauses, distinctive tone/intensity associated with core negative symptoms like flat affect, decreased emotional expression and difficulty controlling speech to express emotion properly (59–65). Given that Espinola et al. (25) found vocal features distinguishing major depressive disorder, schizophrenia and other disorders, our MFCC derived i-vector approach may reveal subtle differences in anhedonic/sad depression symptoms versus alogia/flat affect in schizophrenia. However, further voice difference investigation between depression and schizophrenia is still needed to offer more explanation.

Another possible reason is that the i-vectors catch disorder-relevant information. To extract i-vectors, first the method learns shared case/control information, then removes shared components, yielding i-vectors capturing key individual differences (29, 43). Here, i-vectors captured distinct vocal information for different mental disorders.

Overall, vocal features could provide clinical value in distinguishing depression from selected disorders, and model improvement is helpful, especially for closely related conditions. This analysis establishes a foundation for future studies exploring vocal biomarkers for differential diagnosis of psychiatric disorders.

This research was unable to clearly differentiate between depression and BD. There appears to be substantial symptom overlap between these two conditions. Furthermore, the oscillation between manic and depressive states in BD can be subtle, as demonstrated by Grande et al. (66). Researchers have posited that there might be continued presence of subsyndromal residual symptoms during recovery from major affective episodes in bipolar disorder, and residual symptoms after resolution of a major affective episode indicate that the individual is at significant risk for a rapid relapse and/or recurrence, augmenting the intricacy of symptom presentation even during euthymia (67). Depressive symptoms may have been present in euthymic bipolar patients in this study, confounding diagnostic classification. This observation highlights the complexity of the euthymic bipolar condition. Future research should examine symptom and voice differences between BD phases and depression. A nuanced understanding of markers that distinguish unipolar depression from BD could sharpen diagnostic precision.

This study has limitations. We examined only depression, BD and schizophrenia in a small cultural sample, limiting generalizability. It is worth noting that this study lacks an anxiety disorder group, which also exhibits affective symptoms similar to depression. As the data came from a previous project in China, anxiety diagnoses were not included. Future work will gather systematic data on symptoms, clinical phase, psychological factors like emotion, cognition, and severity in these and other disorders, such as anxiety. This could yield insights into voice differences between depression and other conditions, enhancing the clinical value of vocal biomarkers.

## Conclusion

This research systematically explored the ability of vocal biomarkers to distinguish depression from disorders with similar affective symptoms. Findings suggest vocal features could aid differential diagnosis for depression in clinical practice. Future research should investigate mechanisms by which vocal features differentiate depression and other disorders, and develop more advanced machine learning models so voice can enhance clinical diagnosis.

## Data availability statement

The raw data supporting the conclusions of this article will be made available by the authors, without undue reservation.

## Ethics statement

The studies involving human participants were reviewed and approved by the Institutional Review Board (IRB) of Institute of Psychology, Chinese Academy of Sciences. The patients/participants provided their written informed consent to participate in this study.

## Author contributions

WP was in charge of overall research, data analysis, and drafting the paper. FD was in charge of offering professional guidance and advice for mental illnesses as a clinical specialist. XW helped on data analysis. BH and WZ helped with the analysis and revising the manuscript. TZ was in charge of collecting and organizing the data. All authors contributed to the article and approved the submitted version.

## Funding

This work was supported by the Fundamental Research Funds for the Central Universities (CCNU21XJ021), Knowledge Innovation Program of Wuhan-Shuguang Project (2022020801020288), and the Research Program Funds of the Collaborative Innovation Center of Assessment toward Basic Education Quality (2022-04-030-BZPK01).

## Conflict of interest

The authors declare that the research was conducted in the absence of any commercial or financial relationships that could be construed as a potential conflict of interest.

## Publisher’s note

All claims expressed in this article are solely those of the authors and do not necessarily represent those of their affiliated organizations, or those of the publisher, the editors and the reviewers. Any product that may be evaluated in this article, or claim that may be made by its manufacturer, is not guaranteed or endorsed by the publisher.
